# The effect of temperature on childhood hand, foot and mouth disease in Guangdong Province, China, 2010–2013: a multicity study

**DOI:** 10.1186/s12879-019-4594-y

**Published:** 2019-11-12

**Authors:** Zece Xu, Wenqi Hu, Kedi Jiao, Ci Ren, Baofa Jiang, Wei Ma

**Affiliations:** 1 Department of Epidemiology, School of Public Health, Shandong University, 44 West Wenhua Road, Jinan, Shandong 250012 People’s Republic of China; 2 Qianfoshan Hospital of Shandong Province, 16766 Jingshi Road, Jinan, Shandong 250012 People’s Republic of China; 3 Shandong University Climate Change and Health Center, 44 West Wenhua Road, Jinan, Shandong 250012 People’s Republic of China

**Keywords:** Hand, foot and mouth disease, Two-stage analysis, Distributed lag non-linear model, Multivariate meta-analysis

## Abstract

**Background:**

Hand, foot and mouth disease (HFMD) is a serious infectious disease, which has become a public health problem. Previous studies have shown that temperature may influence the incidence of HFMD, but most only focus on single city and the results are highly heterogeneous. Therefore, a multicity study was conducted to explore the association between temperature and HFMD in different cities and search for modifiers that influence the heterogeneity.

**Methods:**

We collected daily cases of childhood HFMD (aged 0–5 years) and meteorological factors of 21 cities in Guangdong Province in the period of 2010–2013. Distributed lag non-linear model (DLNM) with quasi-Poisson was adopted to quantify the effects of temperature on HFMD in 21 cities. Then the effects of each city were pooled by multivariate meta-analysis to obtain the heterogeneity among 21 cities. Potential city-level factors were included in meta-regression to explore effect modifiers.

**Results:**

A total of 1,048,574 childhood cases were included in this study. There was a great correlation between daily childhood HFMD cases and temperature in each city, which was non-linear and lagged. High heterogeneity was showed in the associations between temperature and HFMD in 21 cities. The pooled temperature-HFMD association was peaking at the 79th percentile of temperature with relative risk (*RR*) of 2.474(95% *CI*: 2.065–2.965) as compared to the median temperature. Latitude was the main modifier for reducing the heterogeneity to 69.28% revealed by meta-analysis.

**Conclusions:**

There was a strong non-linear and lagged correlation between temperature and HFMD. Latitude was strongly associated with the relationship between temperature and HFMD. Meanwhile, it had an effect on modifying the relationship. These findings can conducive to local governments developing corresponding preventive measures.

## Background

Hand, foot and mouth disease (HFMD) is an infectious viral illness which mainly infects children aged 0–5 years old [1]. CA16 and EV71 are the most common pathogenic enterovirus causing HFMD [2]. EV71 may cause severe nervous system syndrome even death [3]. The disease can be infected through several pathways: 1) contacting with infected person’s spittle or blister fluid directly; 2) fecal-oral route; 3) respiratory pathway; 4) contacting with contaminated objects [4, 5]. The incubation of HFMD is about 3–7 days [6]. Pain, fever, bad appetite, small blisters and ulcers on hands, feet, mouth and other parts are the main symptoms. Most children will self-cure after a week, but some may catch more serious symptoms like myocarditis, pneumonedema, meningitis and other syndromes. There is still lacking of effective vaccine to prevent childhood HFMD [7].

HFMD was widespread in the world since the first case reported in New Zealand in 1957. Singapore (1970), the Republic of Korea (2009) and Vietnam (2011) also witnessed outbreaks [4, 8–10]. Epidemic of HFMD in China is serious and brings a heavy burden. Outbreaks of HFMD have occurred in Linyi (2007) and Hong Kong (2010), respectively [11, 12]. In China, HFMD was classified as notifiable disease in 2008 with 28,196 reported cases in China in February 2019 [13, 14]. The prevalence of HFMD has been a critical public health problem and attracted the attention of the government.

Many studies have proved that the incidence of HFMD is related to seasons. Studies in Japan and Finland indicated that seasonal peak occurred in summer and autumn, respectively [16, 17]. What the two studies have in common is that there is only one peak. In contrast, two peaks have been found in some countries with subtropical and tropical climates, such as Taiwan, Hong Kong, Malaysia and mainland China [15, 18–20]. The seasonality of HFMD indicates that HFMD may be associated with meteorological factors [21].

Several researches have confirmed the association between temperature and the incidence of HFMD [7]. The mechanisms maybe that these factors could affect the breeding, growth and transmission of virus and human living habits [22]. In addition, evidence for the temperature-HFMD association has been found by many studies [5, 13, 20, 23, 24], but the results were inconsistent. Studies in Beijing and South Korea showed that the risk increased as temperature rose within a certain high temperature range but declined at extreme high temperature [13, 24]. An analysis in Japan revealed that the increased weekly cases were related to average temperature [16]. Most previous studies were focused on single-site, few papers researched the heterogeneity among different sites and studies.

In this study, we try to fill the gap through a multicity research to explore the heterogenicity among cities and potential effect modifiers. We conducted a two-stage analysis based on 21 cities in Guangdong Province from 2010 to 2013. In addition, distributed lag non-linear model (DLNM) was developed to evaluate the effect of temperature on HFMD in specific-city. Some city-specific characteristics including meteorological factors, geographical factors and socio-economic factors were included to estimate their modifications.

## Methods

### Study area

**Fig. 1:**
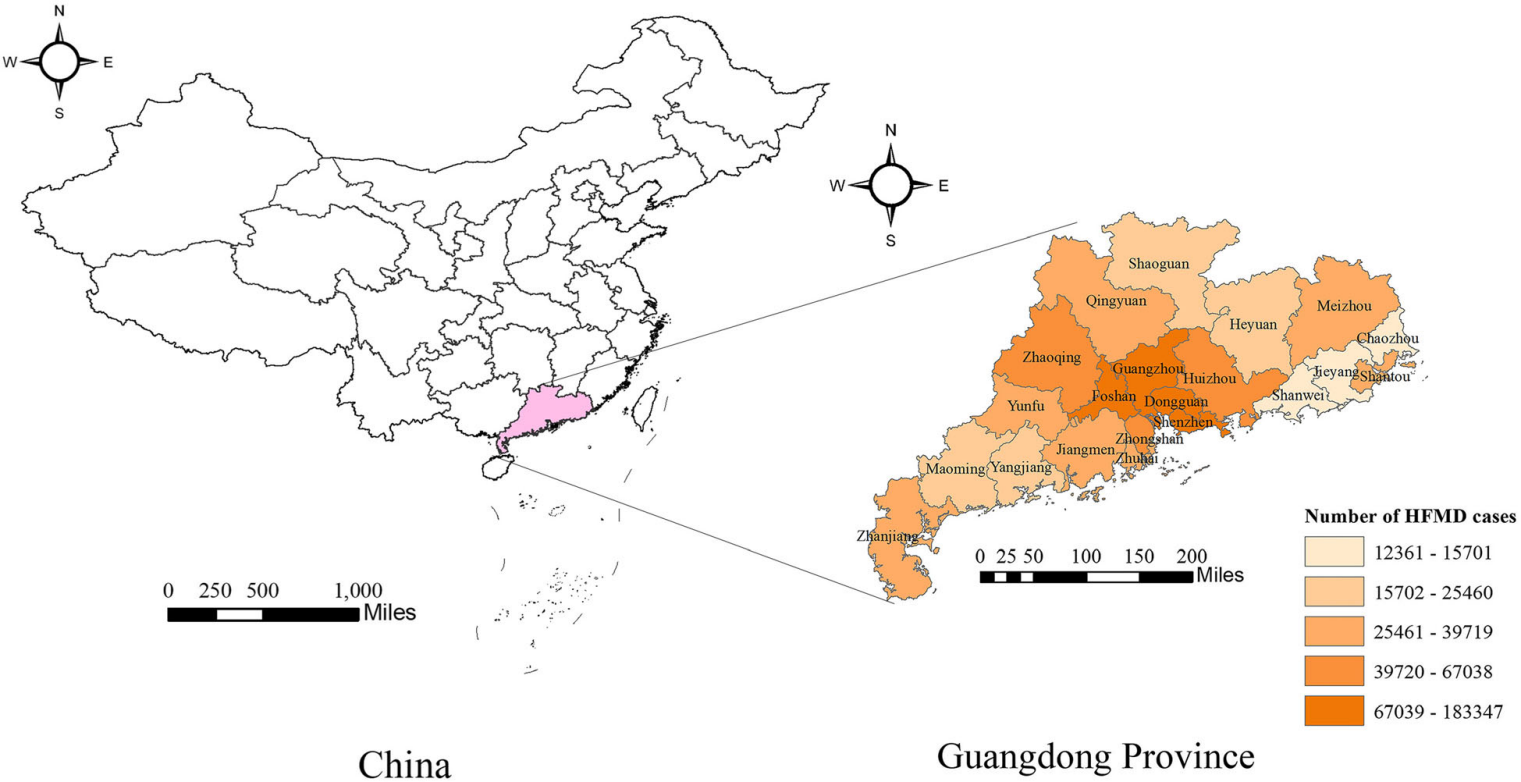
Geographic locations and spatial distribution of HFMD incidence for the 21 cities of Guangdong Province in China, 2010–2013. The map was generated using ArcGIS 10.4 (Environmental Systems Research Institute, Redlands California, America)

Guangdong Province is located in the southern tip of the Chinese mainland coast, with a total of 21 prefecture-level cities and a population density of 621 persons per km^2^ (in 2017: population = 111,690,000 persons; land area = 179,700 km^2^). The province is characterized by subtropical monsoon climate. The annual mean temperature and precipitation is 22.4 °C and 1710.7 mm. The per capita Gross Domestic Product of Guangdong is 80,932 CNY, approximately 1.4 times as much as the national average [25]. Fundamental digital maps of China and Guangdong Province were obtained from the National Earth System Science Data Sharing Infrastructure [26]. HFMD has been prevalent in Guangdong Province for years and become an important disease affecting people’s health. But the incidence varies from city to city. Guangdong Province belongs to subtropical monsoon climate, which is conducive to study the association between temperature and HFMD. All these prompted us to study the epidemiological situation of HFMD in this area. See Fig. 1.

### Data sources

All clinical and laboratory cases reported from 1 January 2010 to 31 December 2013 were obtained from China National Notifiable Disease Surveillance System (NDSS). Some basic information about cases, such as current address, age, gender, the onset data and daily counts of cases in each city were also collected. In this study, we mainly focus on children 0–5 years old because they are more sensitive to HFMD and majority of reported cases were in this age group [1].

Daily meteorological variables, including mean temperature, maximum temperature, minimum temperature, average relative humidity, mean pressure, mean wind speed, precipitation and sunshine hours for the same period were collected from China Meteorological Data Sharing Service System.

Some city-specific variables between 2010 and 2013 of the 21 cities were collected from Guangdong Statistical Yearbook, including meteorological factors (temperature, precipitation, relative humidity and sunshine hours), geographical factors (latitude and longitude), socio-economic factors (GDP, GDP per person, average population, population density, land area).

### Statistical analysis

We adopted a two-stage analytical method in this study. In the first stage, a time-series distributed lag non-linear model (DLNM) with quasi-Poisson regression was applied to quantify the single and cumulative effect of daily mean temperature on daily cases of HFMD in each city. We hypothesized that daily cases followed quasi-Poisson distribution and used Log function to connect to control over-dispersion and extended the lag period to 21 days to research the long-term exposure-response relationship according to the incubation and duration of HFMD. Cross-basis function was selected to show the exposure-lag-response association by applying natural cubic splines for temperature and lag. A natural cubic spline with 7 degrees of freedom (df) was used for time variable to curb the secular trend. The effect of day of week (DOW) was adjusted in the model. Spearman correlation, and collinearity diagnosis were analyzed to explore and control the meteorological confounding factors. Spearman correlation was to analyze the correlation between meteorological factors and the relationship between meteorological factors and the number of HFMD cases. There was severe collinearity if variance inflation factor (VIF) was greater than 10 indicates. Natural cubic spline with 3 df was used for confounders [27]. Formula is as follows:

$$ \mathrm{Log}\left[E\left({Y}_t\right)\right]=\alpha +{\beta}_1 Tem{p}_{t,l}+ NS\left( Hu{m}_{t,l},3\right)+ NS\left( Su{n}_{t,l},3\right)+\cdots + NS\left( Time,7/ year\right)+\gamma Dow+ lag(res) $$

Y_t_: cases on the t day; α: intercept; Temp_t,l_: cross-basic of temperature and time; β_1_: coefficient; Hum_t,l_: relative humidity; Sun_t,l_: sunshine hours; lag(res) refers to the fourth-order lagged variable of the model residual error to control residual autocorrelation. We determined the df of temperature and lag both as 4, according to quasi-akaike information criterion (QAIC), when QAIC was the smallest. All of the meteorological factors included in the model were converted to their percentile. The median of temperature was defined as reference when calculating relative risk (*RR*).

In the second stage, restricted maximum likelihood (REML) and random-effect model was applied in multivariate meta-analysis and the exposure-response relationship of 21 specific cities obtained from the first stage were pooled [28]. To explore the factors influencing exposure-response relationship among different cities, some city-specific variables were included in meta-regression as potential effects modifiers. Wald test was used to test the ability of predictors in explaining heterogeneity among 21 cities. Goodness of fit test for the model was based on Akaike information criterion (AIC) and Bayesian information criterion (BIC). The residual heterogeneity was tested by multivariate extension of Cochran Q test and *I*^2^ statistic (the residual heterogeneity was statistically significant when *P* < 0.05) [29].

Analyses were conducted using R software 3.4.3 with the packages “dlnm” and “mvmeta” and IBM SPSS Statistics 24.0. The difference was considered statistically significant when *P* < 0.05 with two sides.

## Results

Totally 1,105,117 HFMD cases were reported from January 1, 2010 to December 31, 2013, among which 1,048,574 (94.9%) were children aged 0–5 years old. In this study, we mainly focused on children under 5 years old. The average annual incidence in Guangdong Province was 262 per 100,000 persons in the whole population. The male to female sex ratio was 1.71 for childhood cases (range: 1.10–2.10). Daily mean temperature was similar among 21 cities, ranging from 20 to 23.5 °C. More detailed information about 21 cities is shown in supplementary data (see Additional file 1). The majority cases occurred in children were under 1 year old, accounting for 31.2%. HFMD cases were mainly scattered and childcare children, accounting for 83.7% and 16.0% of total cases, respectively. Most cases were between 0 and 3 years old (87.8%), and among them 88.8% were scattered children, but children between 4 and 5 years old were primarily childcare children.

**Fig. 2:**
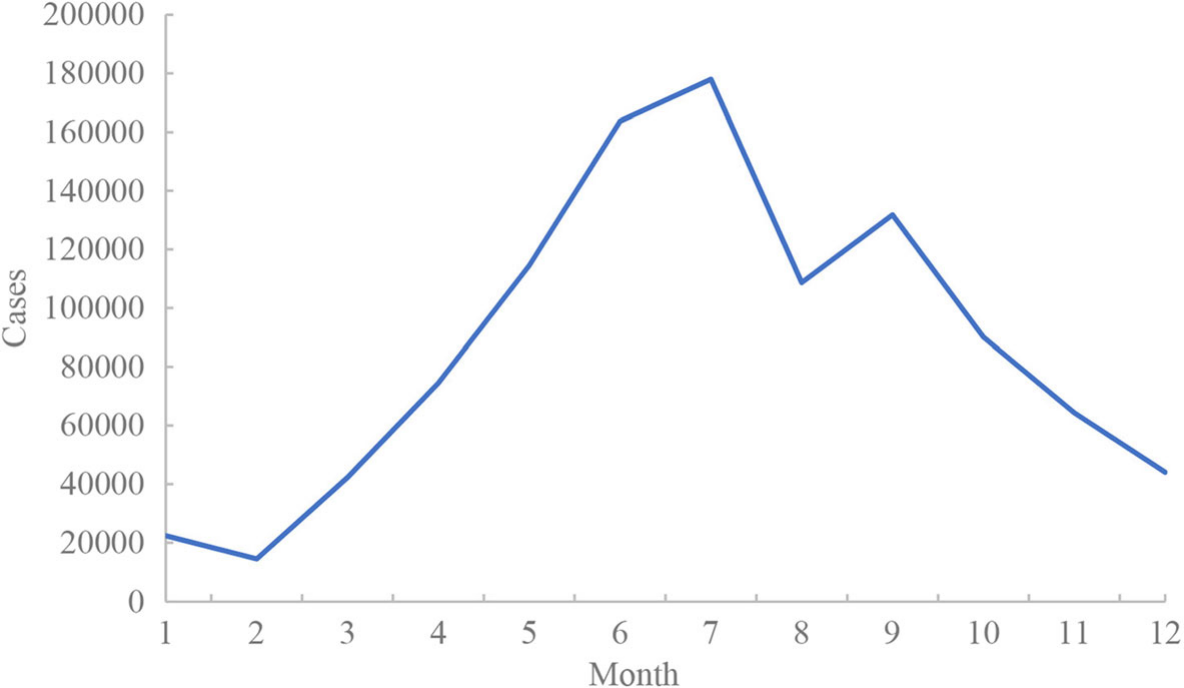
Seasonal distribution of HFMD cases in Guangdong, 2010–2013

Figure 2 showed the seasonal distribution of HFMD cases. Obvious seasonality could be observed with two peaks: the first peak was in late spring and early summer (May to July), and the next appeared in early autumn (September), indicating that meteorological variables might be associated with HFMD.

Table 1 revealed that all the meteorological factors were significantly correlated with daily cases of HFMD. And they were all related to each other, but the correlation coefficient was moderate. In addition, the result of collinear diagnosis showed there was no serious collinearity among meteorological factors for VIF < 10. Consequently, they were all modeled as confounding factors.

**Table 1:** Spearman correlation analysis between HFMD cases and daily meteorological factors

Meteorological factors	cases	precipitation	pressure	wind speed	temperature	humidity	Sunshine hours
cases	1.000						
precipitation	0.077*	1.000					
pressure	−0.201*	− 0.213*	1.000				
wind speed	−0.061*	0.060*	0.090*	1.000			
temperature	0.329*	0.097*	−0.642*	−0.120*	1.000		
humidity	0.099*	0.368*	−0.317*	−0.074*	0.228*	1.000	
Sunshine hours	0.079*	−0.290*	− 0.081*	−0.079*	0.362*	− 0.453*	1.000

*p < 0.05

**Fig. 3:**
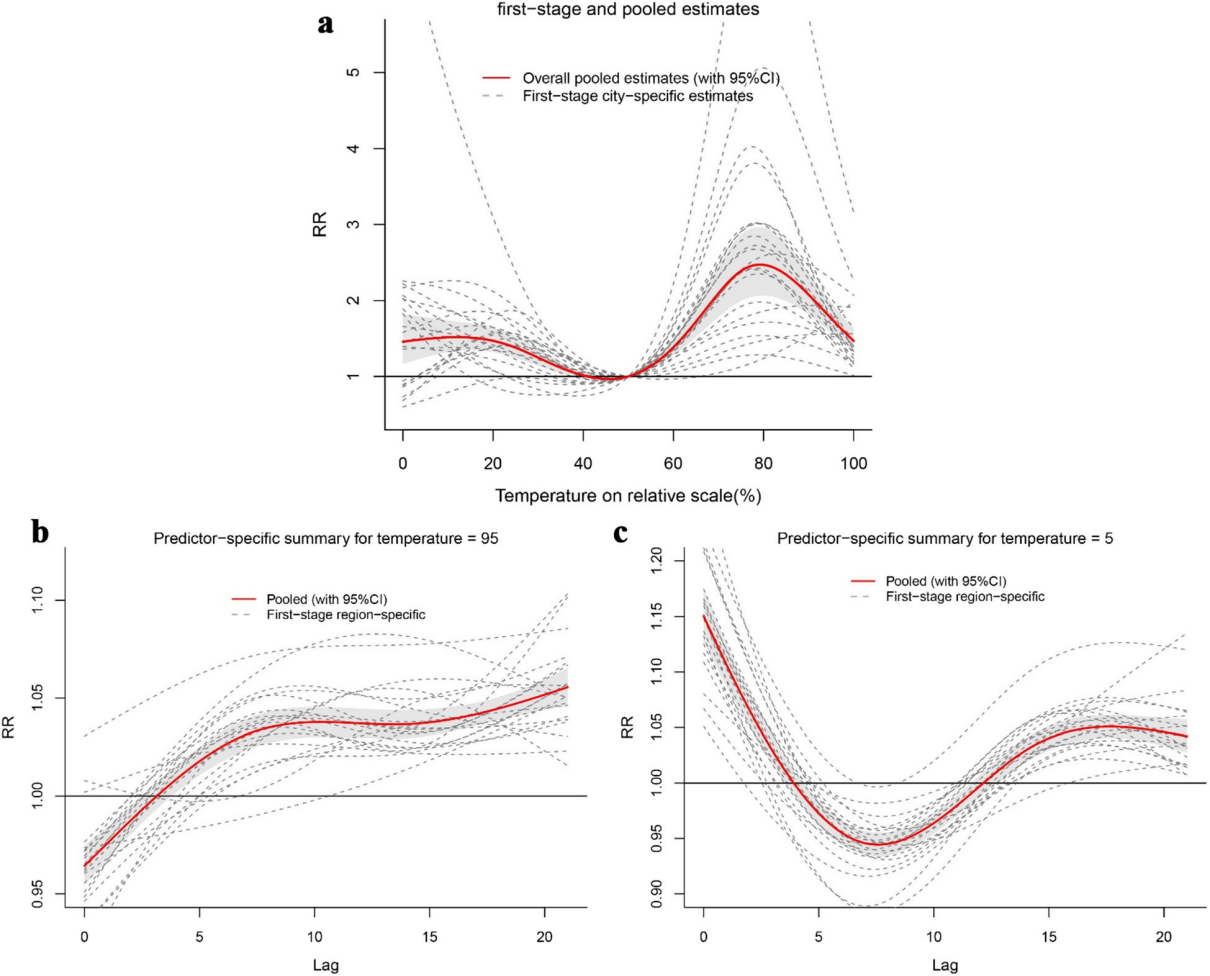
Pooled effects of temperature on HFMD in Guangdong, 2010–2013. The first picture (**a**) shows the overall cumulative effects over lag 0–21 days in 21 cities, the last two pictures describe (**b**, **c**) the pooled effects at predictor-specific (95th and 5th percentile of temperature). The dotted lines represent the different effects of 21 cities, the red line represents the pooled effect and the shaded area is the confidence interval (*CI* with 95%). The median value was reference

Figure 3 showed the exposure-response relationships in each of 21 cities. The results in 21 cities were greatly heterogeneous (Cochran Q test, Intercept-only, *P* < 0.01). The pooled overall cumulative effect revealed that the temperature-HFMD association was nonlinear with an approximately “M” shape. At beginning, the pooled overall cumulative effect was at a slowly rising and then declining level until the temperature reached 41th percentile. After 50th percentile, the risk started to rise until reached the maximum relative risk (*RR* = 2.474, 95% *CI*: 2.065–2.965) at 79th percentile with 50th percentile as reference. Then the *RR* decreased when temperature was higher than that. The results showed that both hot (95th percentile) and cold temperature (5th percentile) could increase risk, and *RR*s were 1.766 (95% *CI*: 1.581–1.973) and 1.494(95% *CI*: 1.253–1.781), respectively. The bottom pictures (b and c) in Fig. 3 showed the association between temperature and HFMD at predictor-specific (95th and 5th percentile), with the same reference (50th percentile), respectively. The risky effect of hot temperature on HFMD appeared later and lasted longer compared with cold temperature.

The results of random-effect meta-analysis (intercept-only) and multivariate meta-regression (with a meta-predictor) in the second stage were showed in Table 2. The heterogeneity among different cities was statistically significant based on the Cochran Q test (Q = 372.07, *P* < 0.001), *I*^*2*^ suggested that 78.50% of heterogeneity was due to actual difference of 21 cities. Residual heterogeneity was still high, although some predictors included in the model could explain a part of heterogeneity, such as land area, latitude, longitude, population density, humidity and sunshine hours according to the results of meta-regression (Wald test *P* value< 0.05). Although we examined the effects of average population, economic factors and precipitation, no evidence of association with the temperature-HFMD relationship was found. Results of multivariate meta-analysis indicated that geographical factors (latitude and longitude) were significantly related to the heterogeneity for decreasing *I*^2^ to 69.28% and 73.48%, respectively, although residual heterogeneity were still significant (Cochran Q test *P* < 0.001).

**Table 2:** Meta-analysis and meta-regression

Meta-predictors	Cochran Q test			*I*^2^	Model fits		Wald test		
	Q	df	*p*	(%)	AIC	BIC	Stat	df	*p*
Intercept-only	372.07	80	< 0.001	78.50	154.49	187.83	–	–	–
Land area	352.81	76	< 0.001	78.46	227.24	269.19	16.39	4	0.003
Latitude	247.36	76	< 0.001	69.28	148.19	190.14	43.51	4	< 0.001
Longitude	286.57	76	< 0.001	73.48	160.06	202.02	30.52	4	< 0.001
Average population	361.42	76	< 0.001	78.98	214.80	256.75	3.91	4	0.418
Population density	342.98	76	< 0.001	77.84	217.13	259.09	15.37	4	0.004
GDP	353.07	76	< 0.001	78.47	234.08	276.03	5.31	4	0.257
GDP per person	364.44	76	< 0.001	79.15	252.13	294.08	4.07	4	0.397
Temperature	316.06	76	< 0.001	75.05	156.28	198.23	20.75	4	< 0.001
Precipitation	364.85	76	< 0.001	79.17	215.11	257.07	2.10	4	0.717
Humidity	287.29	76	< 0.001	73.55	165.65	207.60	18.73	4	< 0.001
Sunshine hours	325.75	76	< 0.001	76.67	192.83	234.78	32.89	4	< 0.001

**Fig. 4:**
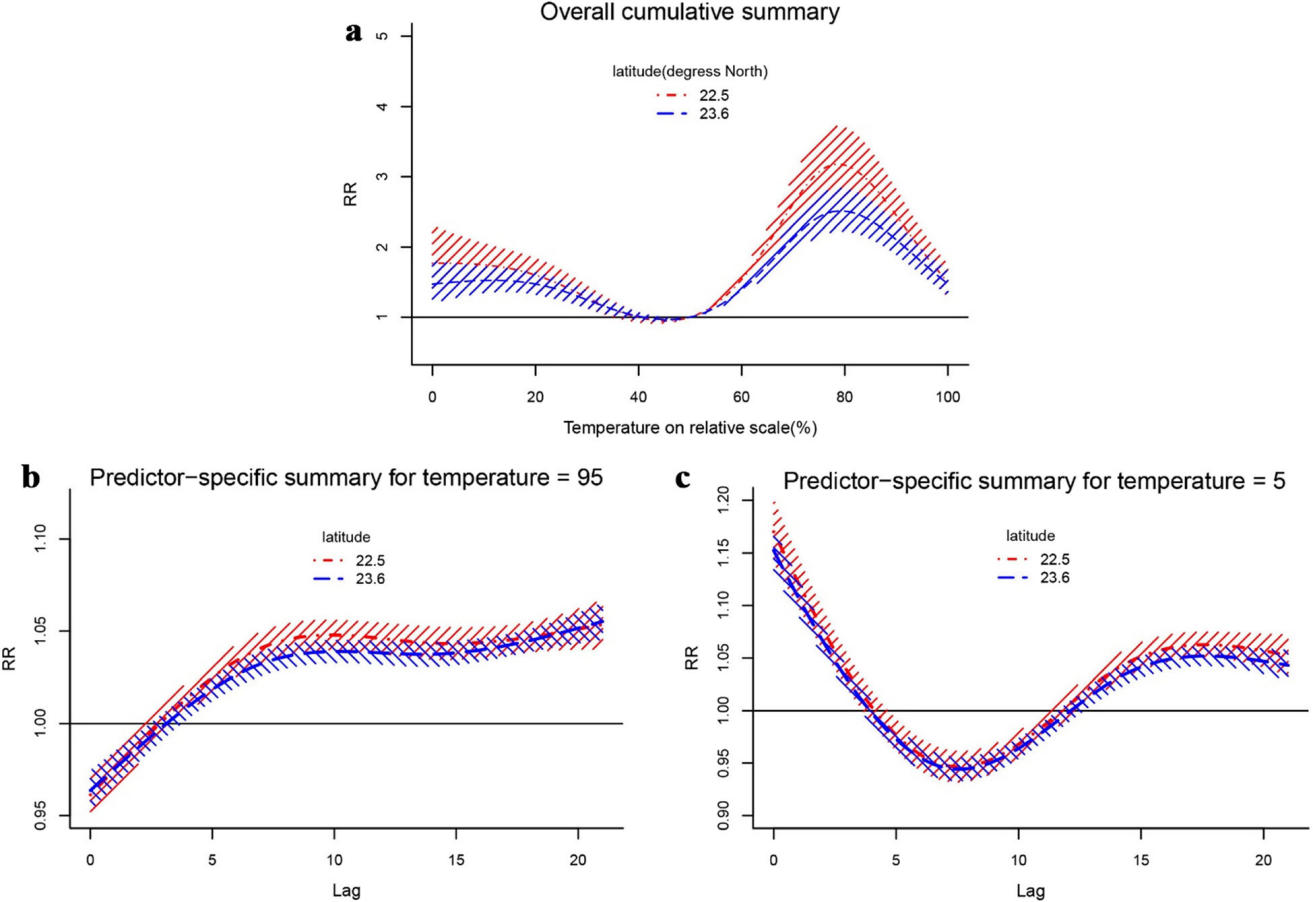
The pooled effects of temperature on HFMD by latitude in 21 cities of Guangdong province, 2010–2013. The first picture shows predictions at 75th (blue line) and 25th (red line) of latitude from meta-regression for overall cumulative summary (**a**), the last two pictures (**b**, **c**) show predictor-specific summary at 95th and 25th of temperature, respectively. The dashed area is 95% confidence interval

Figure 4 illustrated the results of meta-regression with latitude. The effect modification of latitude occurred to both high and low temperature (Wald test *P* < 0.05, Fig. 4b, c). At 95th percentile of temperature, the pooled overall cumulative effects were 1.946 (95% *CI*: 1.701–2.227) and 1.782 (95% *CI*: 1.610–1.972) for 25th and 75th percentile of latitude, respectively (Fig. 4a). The effects were 1.768 (95% *CI*: 1.446–2.163) and 1.504 (95% *CI*: 1.288–1.757) at 5th percentile (Fig. 4a).

In this study, we also explored the temperature-HFMD relationship in various age groups. The children aged 0–5 were divided into 0–3 and 4–5 years old to examine whether the temperature-HFMD curve could be affected by the age group. The results displayed that the curves were similar for different age groups (see Additional file 2 and Additional file 3).

Sensitivity analysis for pooled cumulative effect was conducted by changing the df for temperature from 4 to 6 (see Additional file 4). The result demonstrated that the df we chosen was suitable because exposure-response curve did not significantly change.

## Discussion

HFMD has been a threat to the health of children worldwide. In this study, we conducted a two-stage analysis to investigate the temperature-HFMD relationship in 21 cities and effect modifiers. All cities and cases in Guangdong Province were included, which can fully reflect the incidence in the province. The province is conducive to study the temperature-HFMD association for it is close to Pacific Ocean, the climate of each city is different. The confounding factors vary slightly for all cities in the same province.

The number of cases aged 0–5 was 1,048,574 (accounting for 94.9% of all cases), 87.8% of which was children under three. This is consistent with other studies. For example, some studies observed that children under three were more susceptible to HFMD for lacking of corresponding antibodies [30–32]. Another study showed that the onset risk of infant less than 1 year old is relatively low compared to children 1–3 years old, the possible reason is that they still have antibodies obtained from the mother [33]. The study showed that children aged 4 to 5 are also at high risk of HFMD, which probably because there are a lot of childcare children at this age, and they have more chances to contact with each other and expose to contaminants, making it easier for HFMD to spread. Cases of male children were 1.71 times higher than that of female, which was higher than other studies [1, 34]. The difference can be explained by their genetic level [15], lifestyles and the substances they exposed to. Besides, boys are naturally active and more likely to expose to contaminants.

The number of HFMD cases peaked around May–July because the climatic conditions in this period, such as temperature, humidity, sunshine, are suitable for the survival and reproduction of pathogens. Similar seasonal peaks occur throughout China, but it varies slightly in different areas. For example, the peak of HFMD cases appears May to July in Beijing, April to July in Chengdu and April to June in China [13, 23, 35].

Many studies have explored the effects of meteorological factors on HFMD, among which the influence of temperature has been widely researched [16, 23, 36]. Weekly or monthly morbidity data was used in many literatures, which could not reflect daily incidence and might lead to deviations in the results. Daily cases and meteorological data were used in this study to improve the accuracy of results. Besides, temperature can be expressed in many ways, including daily mean temperature, daily maximum temperature, daily minimum temperature, the last two were widely adopted, which can only reflect extreme condition of daily temperature at a certain point. Daily mean temperature can represent the average level at the same point in two days, and is better than the other two in representativeness [37, 38]. The absolute scale of daily mean temperature in each city was converted into its relative scale to unify temperature and put it into the same model for the daily mean temperature range is different.

The epidemiological triangle emphasizes that pathogens, hosts and the environment are the three elements of disease, and that human health can be maintained unless the balance is broke. Besides, temperature can affect the survival and transmission of pathogen as well as human activities and behaviors [39, 40]. Undoubtedly, it is a key factor in the development of disease. In the first stage, we pooled the overall cumulative effect of 21 cities which showed an approximately “M”-shape which was consistent with the results in other studies (Minhang Distric: a suburban district of Shanghai; Ningbo) [5, 41]. The effect showed a general decline in cumulative risk when temperature was lower than 40th. Possible reason might be that low temperature can impede the reproduction of pathogens and reduce people’s outdoor activities. When temperature was higher than the median value, the *RR* began to rise until reached a peak (2.474 with 95% *CI*: 2.065–2.965), and then decreased when temperature exceeded a certain value. Human will increase outdoor activities when temperature gradually rises, which may increase the chances of children contacting with other children have been infected HFMD virus and they are more likely to come into contact with contaminated food and the rides while playing. The range of temperature in which risk increased may be suitable for the survival of pathogens. All of these will increase the risk of HFMD. The results also showed a decreasing trend when temperature exceed a certain value, which may be explained by a previous study, indicating that temperature and ultraviolet radiation are two factors causing enterovirus inactivation [﻿42﻿]. Therefore, extreme high temperature and ultraviolet radiation are not conducive to the survival of pathogens. In addition, people will reduce outdoor activities and take some protective measures at higher temperature. The city-specific effects at 95th and 25th of temperature pooled in this study revealed that the relative risk came later but continued longer at high temperature compared to low temperature, indicating that the impact of high temperatures on the disease needs to be paid attention to. The pooled effect at 5th percentile of temperature showed that the influence of temperature on HFMD was protective between the lag 4–12, which probably owing to the number of susceptible people reduces over time leading to risk reduction, called “harvesting”. However, the RR increased after the lag 12, probably because of incomplete isolation and contacting to other infected children, which leading to virus spreading again.

Geographical factors, social demographic information, economic condition and meteorological factors were taken into account as potential effect modifiers in this study. The results suggested seven modifiers were related to temperature-HFMD association. Population density can affect the temperature-HFMD relationship by influencing contacting frequency of crowd, utilization of health resources and environmental conditions. Population density can be obtained from land area and average population, so, land area can affect the risk laterally by influencing population density. When a certain population, the smaller the land area, the greater the population density, and the higher the risk of disease. There was no statistical significance between average population and the heterogeneity, which indicated that the real factor affecting the onset risk was population density rather than average population. Economic condition has been identified as a modifier in a previous study [43], but it was not confirmed in this study which may due to there is no significantly difference at economic level among 21 cities. Meteorological factors were considered as potential effect modifiers, though they have been controlled as confounding factors, because meteorological factors might affect the reproduction and living environment of pathogens. Humidity and sunshine hours were confirmed to be effect modifiers in this study. The reason might be that humidity may influence the survival and reproduction of pathogens, and behaviors of host. A systematic review revealed that the *RR* of HFMD increased by 1% (95% *CI*: 1.00–1.02) for every 1% increase in humidity [7]. Ultraviolet radiation could affect the temperature-HFMD association by influencing survival of pathogens to inactivated [﻿42﻿]. In this study, we found that precipitation was not the effect modifier, probably because it distributed evenly in each city. In this study, latitude and longitude were found the most two important effect modifiers which was in agreement with a study including 143 cities of mainland China [36]. The sensitivity to temperature varies for people living in different latitude and longitude, such as, people in low latitude and high longitude are more sensitive to temperature changes. The modification of latitude was slightly higher than that of longitude (*I*^*2*^: 69.28% for latitude, 73.48% for longitude). Figure 4 showed the results of meta-regression with latitude, indicating that people were more sensitive to temperature at lower latitude. Because cities located in lower latitude usually have tropical climate with temperature higher than others and are close to the Pacific Ocean, which will calm them down and increase their sensitivity to temperature in a degree [44]. Significantly, there is still a large part of heterogeneity that cannot be explained by modifiers obtained in the study, suggesting that there are other modifiers that have not been collected, such as air conditioning usage, utilization of medical services, vegetation coverage, preventive awareness and measures, etc.

The study has limitations that should be considered. The method used in this study was ecological study, the relationship between temperature and HFMD could only be observed at the level of crowd, rather than individuals, which might lead to bias. In this study, latitude only explained a part of heterogeneity, there were many other modifiers need to be collected. The results were only applicable to Guangdong Province but failed to extend to other regions. Further studies in different climate sites are needed.

## Conclusion

As severe illness in Guangdong Province, China, HFMD mainly affect children under five. This study verified a non-linear and lagged correlation between temperature and HFMD. We found that there was a great heterogeneity in the temperature-HFMD relationship among 21 cities and latitude had an effect on modifying the relationship. However, further researches are still needed to explore specific spreading mechanism in cities locating in different latitude and effect modifiers. This study can help local health departments provide early warnings to control the risk before reaching its peak. More attention should be paid to the prevention of high temperature because it has a long lag period and preventive measures should be strengthened for people living in lower latitude areas.

## Supplementary information

**Additional file 1.** Meteorological factors and HFMD cases in Guangdong Province, 2010–2013.

**Additional file 2.** The pooled effects of temperature on HFMD aged 0–3 years old.

**Additional file 3.** The pooled effects of temperature on HFMD aged 4–5 years old.

**Additional file 4.** Sensitivity analysis.

## Data Availability

The datasets used in this study are available from the corresponding author on reasonable request.

## Abbreviations

AIC: Akaike information criterion
BIC: Bayesian information criterion
CA16: Coxcackie virusA16
*CI*: Confidence interval
df: Degrees of freedom
DLNM: Distributed lag non-linear model
DOW: Day of week
EV71: Human enterovirus 71
GDP: Gross Domestic Product
HFMD: Hand, foot and mouth disease
NDSS: Notifiable Disease Surveillance System
QAIC: Quasi-akaike information criterion
REML: restricted maximum likelihood
*RR*: relative risk
VIF: Variance inflation factor
