# Supplementary material for: The effect of temperature on childhood hand, foot and mouth disease in Guangdong Province, China, 2010–2013: a multicity study
Source: BMC Infect Dis. 2019 Nov 12;19:969. doi: 10.1186/s12879-019-4594-y (PMC6852944; doi:10.1186/s12879-019-4594-y)
Supplement: Supplementary file 1 — Additional file 1. Meteorological factors and HFMD cases in Guangdong Province, 2010–2013. [file 12879_2019_4594_MOESM1_ESM.docx]

| Meteorological factors and HFMD cases in Guangdong province, 2010-2013 | | | | | | |
| --- | --- | --- | --- | --- | --- | --- |
| City | Temperature | | | | Cases | Sex  ratio |
|  | (℃, mean±sd) | P_5_ | P_50_ | P_95_ |  |  |
| Chaozhou | 21.7±5.7 | 11.3 | 22.8 | 29.1 | 12361 | 1.85 |
| Dongguan | 22.5±6.0 | 10.8 | 24.2 | 29.8 | 107060 | 1.78 |
| Foshan | 21.8±6.2 | 10.1 | 23.2 | 29.6 | 119629 | 1.70 |
| Guangzhou | 21.8±6.2 | 10.1 | 23.2 | 29.6 | 183348 | 1.70 |
| Heyuan | 20.9±6.7 | 8.2 | 22.5 | 29.2 | 21003 | 1.76 |
| Huizhou | 22.6±6.2 | 10.5 | 24.0 | 30.0 | 67039 | 1.61 |
| Jiangmen | 22.7±5.8 | 11.4 | 24.2 | 29.6 | 36905 | 1.68 |
| Jieyang | 21.7±5.7 | 11.3 | 22.8 | 29.1 | 15701 | 1.89 |
| Maoming | 22.9±5.5 | 12.2 | 24.6 | 29.4 | 23645 | 1.96 |
| Meizhou | 21.4±6.5 | 9.1 | 22.9 | 29.6 | 39719 | 1.65 |
| Qingyuan | 20.7±7.0 | 8.0 | 22.3 | 29.6 | 32789 | 1.87 |
| Shantou | 22.1±5.7 | 12.3 | 23.1 | 29.4 | 30569 | 1.87 |
| Shanwei | 22.4±5.5 | 12.6 | 23.5 | 29.2 | 14103 | 1.93 |
| Shaoguan | 20.0±7.3 | 6.8 | 21.5 | 29.2 | 19817 | 1.63 |
| Shenzhen | 23.0±5.6 | 12.1 | 24.4 | 29.6 | 97522 | 1.67 |
| Yangjiang | 22.4±5.6 | 11.4 | 23.7 | 29.0 | 25461 | 1.89 |
| Yunfu | 22.1±6.3 | 9.7 | 23.9 | 29.7 | 32671 | 1.10 |
| Zhanjiang | 23.5±5.3 | 13.1 | 24.9 | 29.7 | 30442 | 2.10 |
| Zhaoqing | 22.0±6.5 | 9.7 | 23.6 | 30.1 | 49365 | 1.91 |
| Zhongshan | 22.5±6.0 | 10.8 | 24.1 | 29.7 | 50227 | 1.60 |
| Zhuhai | 22.9±5.7 | 12.1 | 24.2 | 29.8 | 39198 | 1.69 |
| Total | 22.1±6.1 | 10.5 | 23.5 | 29.5 | 1048574 | 1.71 |
