# Supplementary figures and images for: The effect of temperature on childhood hand, foot and mouth disease in Guangdong Province, China, 2010–2013: a multicity study
Source: BMC Infect Dis. 2019 Nov 12;19:969. doi: 10.1186/s12879-019-4594-y (PMC6852944; doi:10.1186/s12879-019-4594-y)

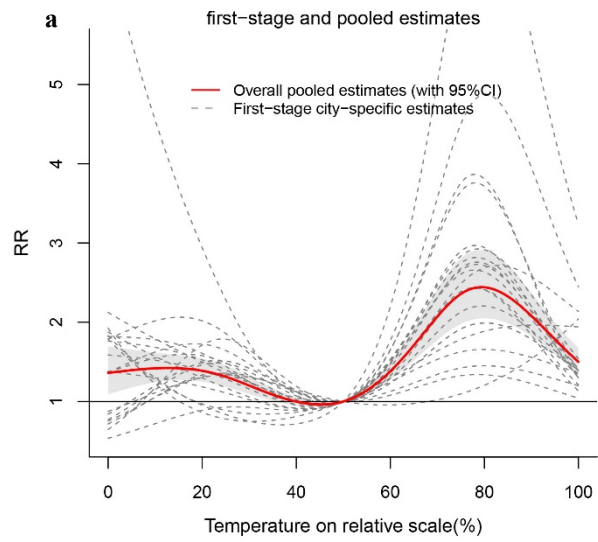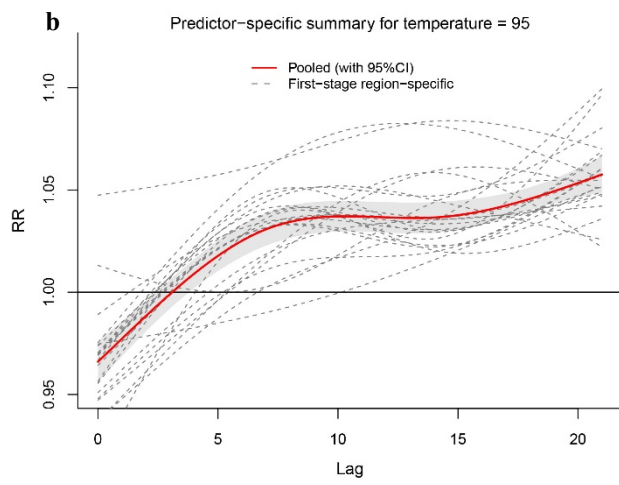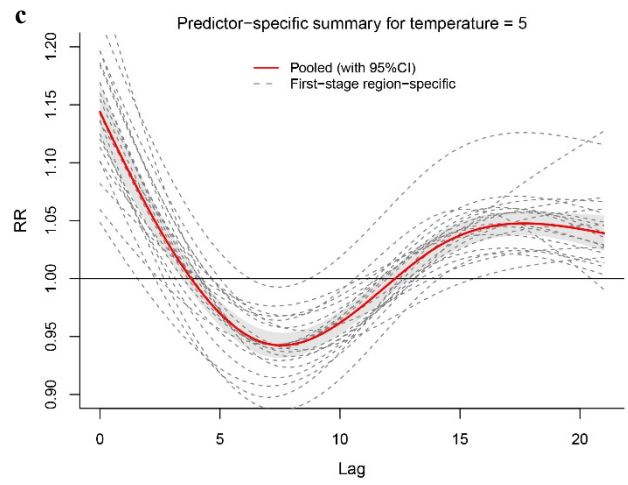

Supplement: Supplementary file 2 — Additional file 2. The pooled effects of temperature on HFMD aged 0–3 years old. [file 12879_2019_4594_MOESM2_ESM.pdf]

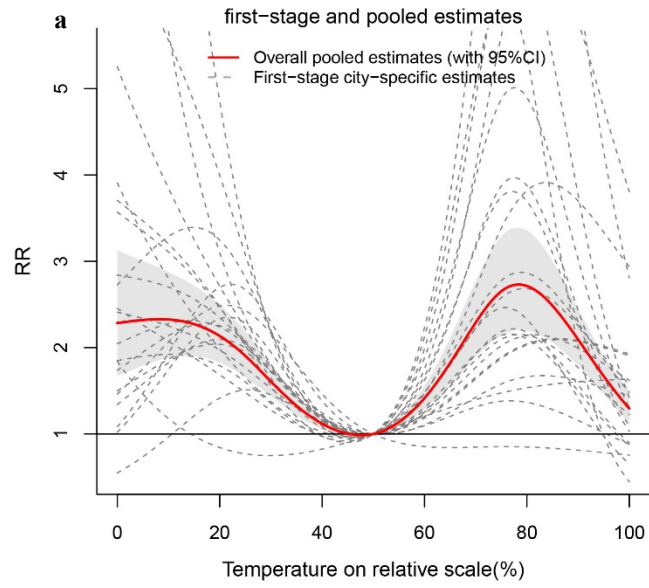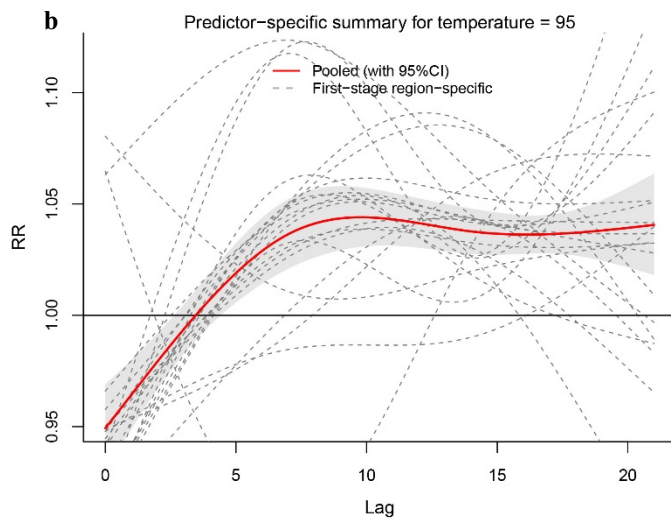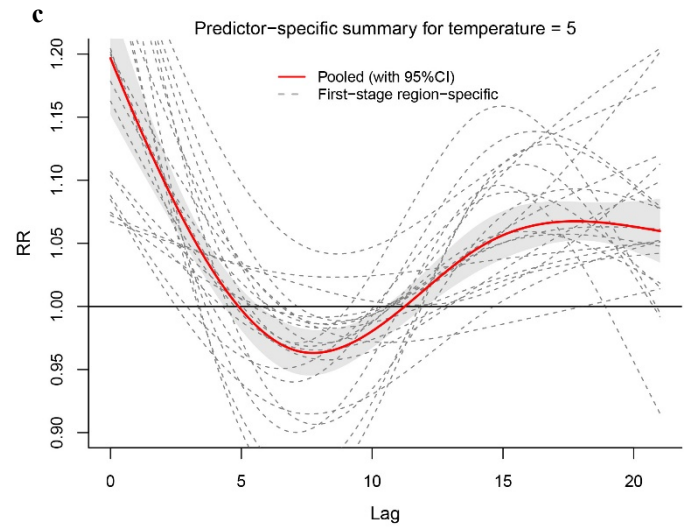

Supplement: Supplementary file 3 — Additional file 3. The pooled effects of temperature on HFMD aged 4–5 years old. [file 12879_2019_4594_MOESM3_ESM.pdf]

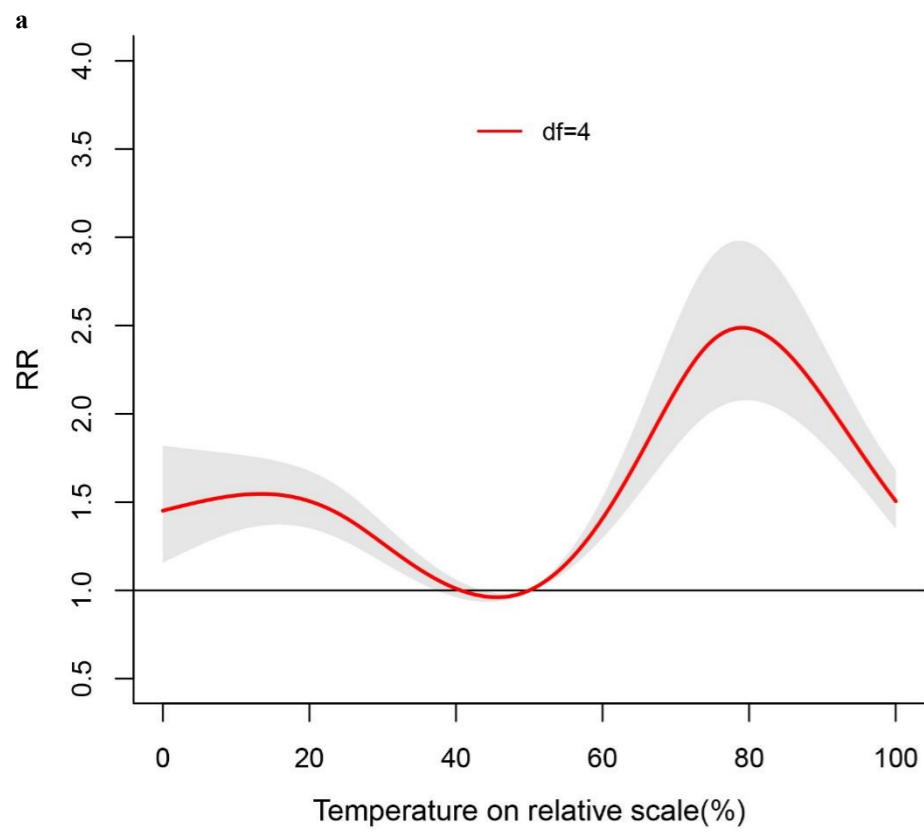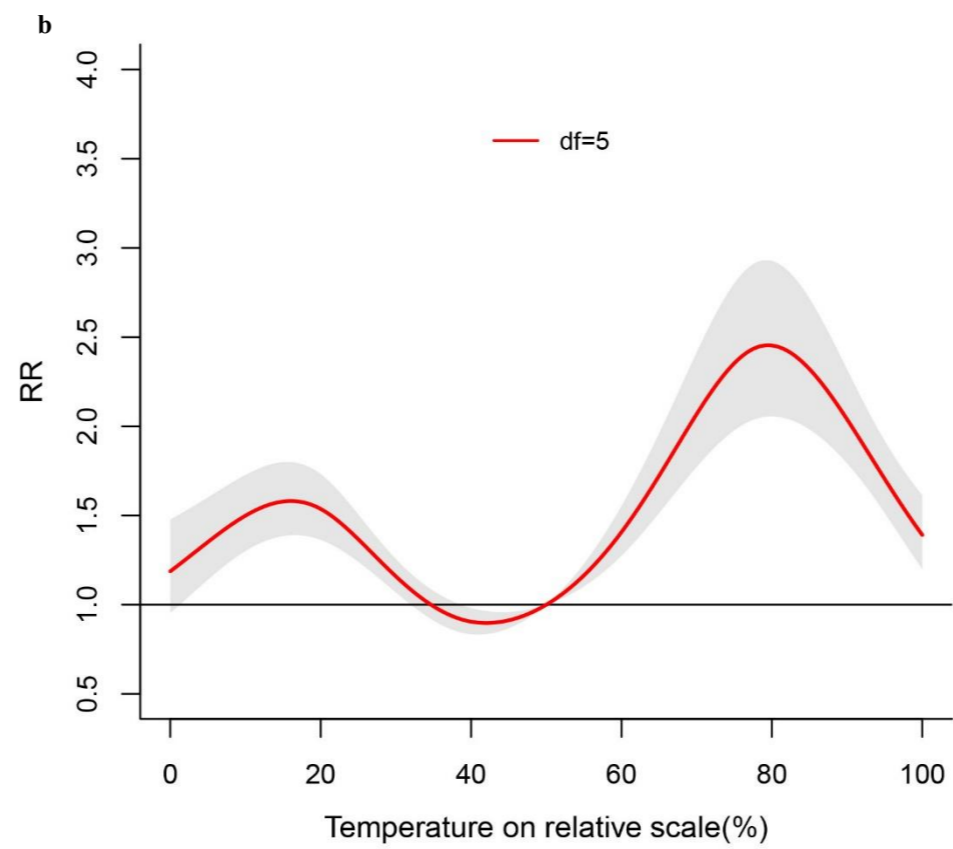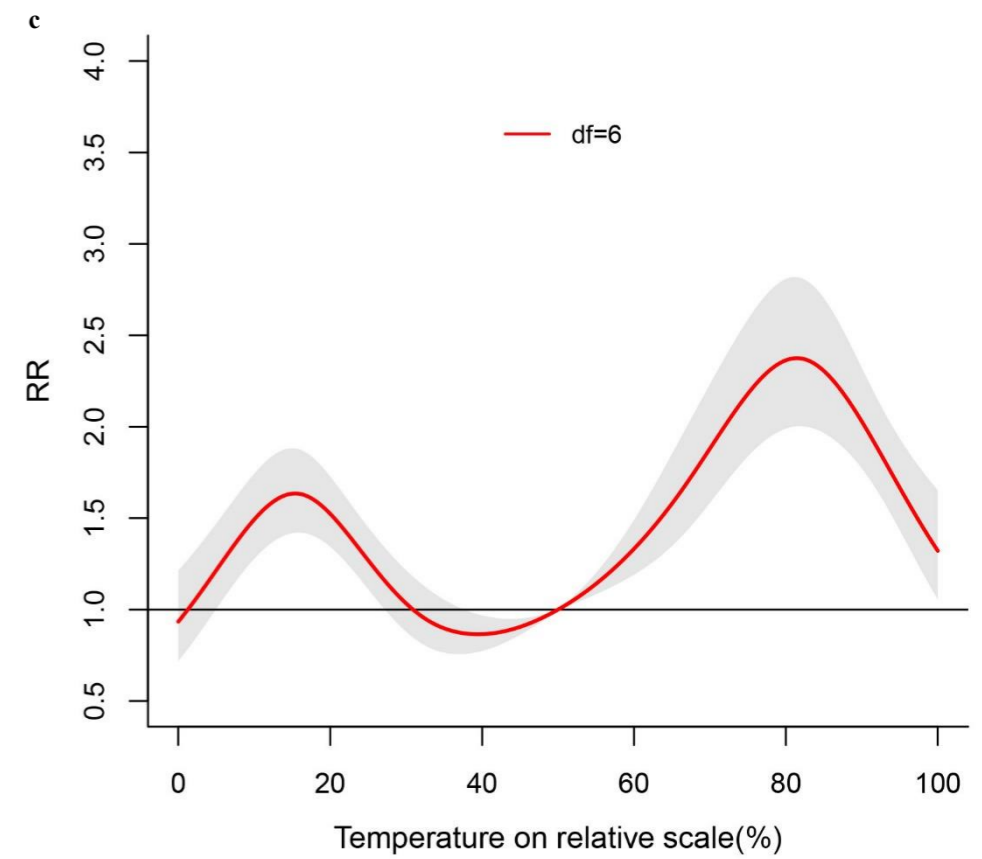

Supplement: Supplementary file 4 — Additional file 4. Sensitivity analysis. [file 12879_2019_4594_MOESM4_ESM.pdf]
